# Eight Weeks of Sprint Interval Training With Adding Caffeine Ingestion Leads to Greater Improvements in Health-Related Physiological Parameters in Obese Male Adults

**DOI:** 10.33549/physiolres.935767

**Published:** 2026-06-01

**Authors:** Jie ZHANG, Qingwei SONG

**Affiliations:** 1 Department of Physical Education, Zhumadian Preschool Education College, Zhumadian, Henan, China; 2 Big Health Collaborative Innovation Institute, Tianjin University of Commerce, Tianjin, China

**Keywords:** Interval intervention, Supplementation, Obesity, Health

## Abstract

The aim of this research was to examine the effects of an 8-week sprint interval training (SIT) intervention alongside caffeine (CAF) supplementation on the physiological adaptations related to the health of obese men. Thirty obese men volunteered to take part in this research and were randomly assigned to three distinct groups: Caffeine plus SIT (CAF+SIT, 6 mg·kg^−1^ body mass, n=10), Placebo plus SIT (PL+SIT, 6 mg·kg^−1^ body mass in cellulose, n=10), and Control (CON, n=10). The participants in the caffeine and placebo groups ingested their supplements three times weekly, one hour prior to each SIT session over a period of eight weeks. Lower body strength, and cardiorespiratory fitness as well as body fat were assessed at baseline and following the intervention. Additionally, blood samples for evaluating fasting glucose and lipid profiles were collected 48 h before and after the SIT intervention. Both the CAF+SIT and PL+SIT groups demonstrated notable changes (p<0.05) in the assessed variables after the 8-week intervention period. Furthermore, the CAF+SIT group exhibited significantly greater adaptive responses (p<0.05) compared to the PL+SIT group in terms of fat mass reduction (Standardized mean difference [SMD]=−0.58), strength improvement (SMD=0.36), and cardiorespiratory fitness enhancement (SMD=0.41), as well as modifications in fasting glucose (SMD=−0.57), HDL (SMD=0.18), LDL (SMD=−0.80), cholesterol (SMD=−0.53), triglyceride (SMD=−0.48) and non-HDL (SMD=−1.14) following the intervention. The administration of CAF prior to SIT led to more significant decreases in fat mass and enhancements in strength and cardiorespiratory fitness. Additionally, it resulted in more notable alterations in fasting glucose and lipid profiles among obese men, thereby elucidating the beneficial effects of CAF when combined with SIT to enhance men’s health.

## Introduction

According to the World Health Organization, the prevalence of obesity has progressively increased since 1975, leading to its classification as a global pandemic [1]. Although dietary modification remains the most effective strategy for reducing body weight [2], structured exercise training represents a critical component of comprehensive weight management and overall health optimization [3]. Current guidelines recommend engaging in at least 150 min of physical activity per week to elicit clinically meaningful reductions in body weight and enhance general health outcomes [3].

Various exercise training modalities have been shown to improve health outcomes in both men, women, and animal models [4–6]. Among these, running-based sprint interval training (SIT) has emerged as an effective strategy for promoting cardiometabolic health [7]. Traditional SIT protocols typically consist of 20–30 s bouts of maximal-effort running or cycling, which may impose substantial mechanical and physiological stress, thereby limiting feasibility for the general population, particularly obese men [8]. In contrast, recent evidence indicates that shorter sprint bouts (<10 s) may provide a more time-efficient and tolerable training approach, eliciting lower perceived exertion and greater exercise enjoyment [8,9] compared with longer interval formats. Notably, short duration of SIT protocols has been associated with significant improvements in maximal oxygen uptake, reductions in body fat, and favorable modulation of lipid profiles [9,10].

In addition to the well-documented effects of exercise training on fat reduction and overall health [3,4], incorporating nutritional supplementation has been shown to potentiate the physiological adaptations induced by exercise interventions [2]. Among these, caffeine (CAF) is well recognized for its thermogenic properties, promoting increased fat oxidation [11] and facilitating weight regulation through elevated secretion of catecholamines such as noradrenaline and dopamine [12]. Moreover, pre-exercise CAF ingestion has been demonstrated to enhance muscular strength and maximal oxygen uptake while concurrently improving lipid metabolism and supporting weight management in individuals with obesity [13–22].

To optimize training-induced adaptations in obese men, CAF supplementation within the range of 3–9 mg·kg^−1^ body weight has been recommended [18,23]. However, recent evidence suggests that a dose of approximately 6 mg·kg^−1^ body weight represents an optimal threshold for enhancing both exercise performance and physiological responses in male populations [19]. Accordingly, careful individualized dosage selection [24] is essential to maximize health-related benefits while minimizing potential adverse effects associated with higher intakes such as gastrointestinal distress, anxiety, cardiac excitability, or sleep disruption [18,19,23]. Consequently, combining a moderate CAF dose of 6 mg·kg^−1^ body weight with SIT may constitute an effective strategy to elicit favorable physiological adaptations and improve overall health outcomes in obese men. However, the potential synergistic effects of CAF supplementation and SIT on health-related adaptations in obese men remain poorly understood. Specifically, the influence of prolonged CAF intake combined with SIT protocols has yet to be systematically investigated. Therefore, the present study aims to examine the effects of pre-exercise CAF ingestion over an 8-week period on fasting blood glucose, lipid profiles, muscular strength, cardiorespiratory performance, and body fat composition in obese men. We hypothesized that chronic pre-exercise CAF supplementation combined with SIT would produce greater improvements in fasting glucose levels, lipid profiles, physical performance, and body fat reduction compared with SIT alone over the 8-week intervention period.

## Materials and Methods

### Ethics approval

Informed consent was obtained from all participants prior to their inclusion in the study. Ethical approval was granted by the Research Ethics Committee of the University’s Institutional Review Board (Approval No. 2025/AA457951GG).

### Participants

Thirty obese adult males (BMI>30 kg/m^2^) with sedentary fitness profiles – defined by engagement in approximately one 60-minute walking session per week and low to moderate intensity resistance training – voluntarily participated in the investigation. Participants were randomly allocated into three equivalent groups: CAF combined with SIT (CAF + SIT; n = 10), placebo combined with SIT (PL + SIT; n = 10), and a non-training control group (CON; n=10) (Table 1). Prior to participation, all volunteers were required to meet the following inclusion criteria:

- Absence of any previous injuries to the lower extremities [25].
- Clearance for training following comprehensive health screening to rule out any musculoskeletal, neurological, or orthopedic conditions that could impair exercise performance or participation.
- No current or recent (within the previous six months) use of pharmacological or therapeutic interventions for obesity management, nor the presence of chronic diseases, endocrine disorders, or diabetes mellitus, as verified by an internal medicine specialist.
- Completion of a detailed health-history questionnaire during recruitment to assess the use of nutritional, pharmaceutical, and hormonal supplements to ensure adherence control.
- Classification as low habitual caffeine (coffee, tea, ….) consumers – defined as consuming less than 50 mg of caffeine per day over the preceding two months – based on the methodology described by Filip *et al*. [26] and assessed using a modified version of the validated caffeine consumption questionnaire developed by Bühler *et al*. [27].
- Participants were also confirmed to be non-smokers. Detailed records of smoking history and current smoking status were maintained.

Based on these eligibility criteria, 18 of the initial 48 volunteers were excluded, resulting in a final cohort of 30 participants who met all inclusion requirements and were enrolled in the study.

### Sample size estimation and study approval

The G*Power software (version 3.1.9; Universität Düsseldorf, Germany) was employed to determine the optimal sample size, using an effect size of 0.21, a statistical power (1-β) of 0.95, and a significance level (α) of 0.05 for F-tests corresponding to a mixed design ANOVA (between and within subject factors). These parameters were derived from a previous investigation assessing the effects of CAF supplementation and high intensity interval training on health-related outcomes in obese men [13]. The analysis indicated that a minimum of eight participants per group was required to achieve adequate statistical power. To mitigate the potential dropout of participants, the sample size was adjusted to ten participants per group. Considering a total of 30 men, the randomization process was executed using a random number generator (i.e., simple randomization) on a computer in a 1:1:1 ratio for the CAF+SIT, PL+SIT, or CON groups (n=10 each), resulting in group assignments that were unpredictable for both authors and participants and based purely on chance. All participants received comprehensive information about the potential risks, benefits and discomforts related to the study and were also obligated to sign informed consent forms. The study protocol was reviewed and approved by the University Ethics Committee (Approval No. 2025/AA457951GG) and conducted in accordance with the latest revision of the Declaration of Helsinki for research involving human participants.

### Study design

**Fig. 1:**
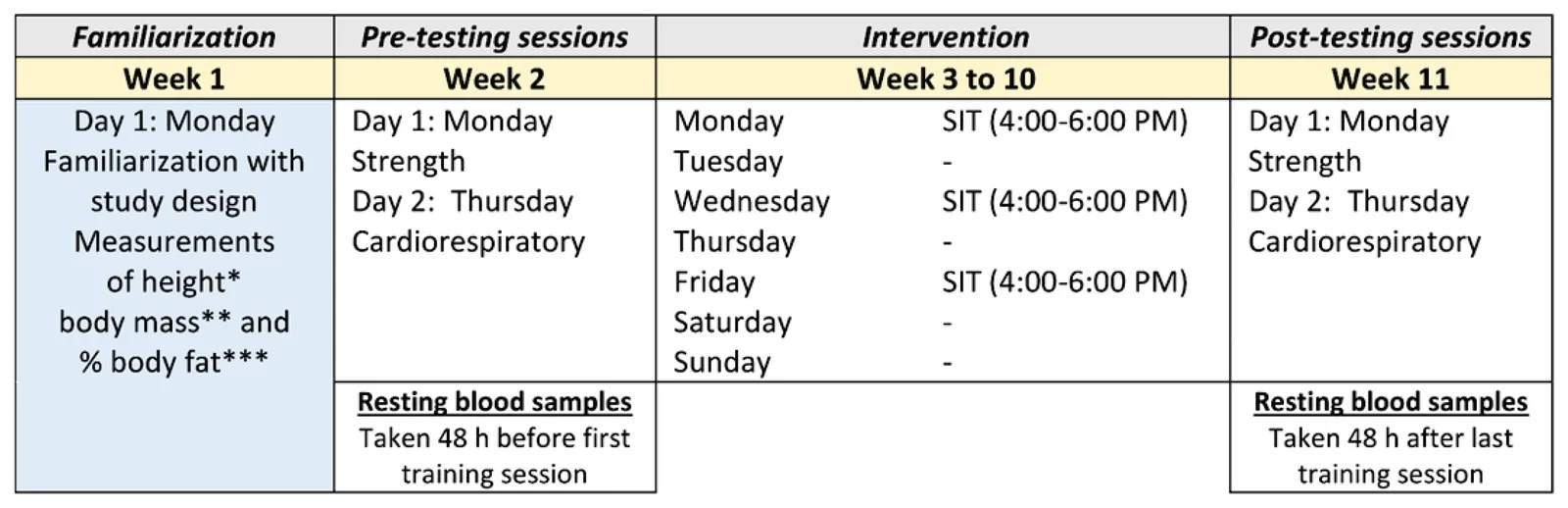
Study design.

The study employed a pre-post experimental design spanning 11 weeks, comprising one week of familiarization with the experimental protocol and testing procedures, one week of baseline assessments, eight weeks of intervention, and one week of post-testing (Fig. 1). During the intervention phase, participants in the CAF group received caffeine supplementation at a dose of 6 mg·kg^−1^ body mass, whereas those in the PL group were administered a cellulose placebo of equivalent dosage. Both supplements were provided one hour before each SIT session over the 8-week period. Evaluations of muscular strength, cardiorespiratory fitness, and body fat (Bioelectrical Impedance Analysis, Tanita BC-551, Japan) were conducted in the afternoon sessions to control for circadian variation. Venous blood samples were drawn in the morning (i.e., 8–9 AM), 48 h before the first and 48 h after the final SIT session, ensuring adequate recovery and the elimination of acute exercise effects. Participants were instructed to abstain from strenuous exercise, maintain their habitual diet for 24 h before testing, and refrain from caffeine and alcohol consumption. Throughout the intervention, they were further advised to preserve consistent lifestyle and dietary habits to minimize confounding influences.

### Caffeine supplementation

During the 8-week intervention, participants allocated to the CAF+SIT and PL+SIT groups ingested either caffeine (6 mg·kg^−1^ body mass; 100 % purity; Bulk Powders, UK) or an inert placebo consisting of encapsulated cellulose one hour before each training session [18,19,23]. All capsules were consumed with approximately 100 ml of juice. To preserve double-blind conditions, no labeling indicated capsule contents, and the visual and gustatory characteristics of the PL were indistinguishable from those of the CAF supplement. Supplements were distributed weekly, and participants were instructed to consume the assigned capsules before each SIT session. At the end of each week, they were required to return all distributed packets – used or unused – to monitor compliance.

### Testing procedures

#### Strength and cardiorespiratory fitness

Lower-body strength performance was assessed using the horizontal leg-press exercise (Force Fitness, UK). The testing procedure followed the protocol described by Kraemer and Fry [28]. Before strength evaluation, participants completed a standardized warm-up consisting of 5 min of light running followed by 5 min of dynamic stretching. During testing, the load was progressively increased across consecutive trials until participants were unable to complete five repetitions with proper technique and full range of motion. The achieved five-repetition maximum (5RM) was then used to estimate the one-repetition maximum (1RM) according to the equation: 1RM = weight (kg)/1.0278 - (5RM × 0.0278) [29]. This indirect estimation was employed to reduce the risk of musculoskeletal strain associated with direct 1RM testing in obese individuals. All tests were supervised by certified researchers, and spotters were positioned beside the machines to ensure participant safety and protocol validity. Participants completed an incremental exercise test to assess cardiorespiratory fitness following a standardized warm-up consisting of 10 min of stretching and 5 min of treadmill running (Technogym, Italy). The protocol began at a speed of 3 km·h^−1^ and was progressively increased by 2.5 % every 2 min until volitional exhaustion, defined as the point where the participant could no longer continue the exercise due to an overwhelming subjective feeling of fatigue. The total exercise duration (in minutes) was recorded and used to estimate peak oxygen uptake (VO_2peak_) using the predictive equation: VO_2peak_ = 1.444 (Time) + 14.99 [30].

#### Blood sampling and analysis

Venous blood samples (15 ml) were drawn from the antecubital vein into plain, evacuated tubes to determine resting concentrations of lipid profiles and fasting blood glucose. Samples were allowed to clot at room temperature for approximately 25–30 min and subsequently centrifuged at 1,500× g for 10 min. The separated serum was aliquoted into microtubes and stored at −80 °C until biochemical analysis. To minimize circadian influences on parameters, all samples were obtained following a 10-hour overnight fast and 8 h of sleep between 08:00 and 09:00 AM. Serum concentrations of total cholesterol, HDL, LDL, and triglycerides were determined using the photometric endpoint method with commercially available enzymatic assay kits (Novus Biologicals, USA) on automated analyzers (Hitachi^®^, models 704 and 902, Japan). Fasting blood glucose was quantified *via* ELISA (Eagle Biosciences, USA). In addition, non-HDL was calculated as follow: non-HDL = cholesterol - HDL. The intra-assay coefficient of variation (CV) was calculated for each analyze and maintained below 5 %, confirming acceptable analytical precision.

#### SIT intervention

All participants maintained their habitual physical activity patterns throughout the study period. The participants in the CAF and PL groups completed a SIT program, while the CON group abstained from any structured exercise program and continued with their normal daily activities. Each SIT session comprised two sets of 10 repetitions of 5-second all-out running sprints, interspersed with 25 s of active recovery. A 3-minute rest interval, including walking and light stretching, was provided between sets to ensure recovery. The SIT sessions were performed three times weekly (Monday, Wednesday, and Friday) over an 8-week period [8,31]. Each session began with a standardized 10-minute warm-up (5 min of light running followed by 5 min of dynamic stretching and ballistic movements) and concluded with a 10-minute cool-down period. All training was conducted on a wooden court surface under controlled environmental conditions (27–28 °C, 45–55 % relative humidity) and was supervised by a certified strength and conditioning coach together with a research team member to ensure full compliance and correct exercise execution.

#### Physical activity and diet controls

All participants were instructed to maintain their habitual dietary and physical activity routines throughout the study. Prior to baseline testing, each participant recorded and analyzed their dietary intake over three consecutive days [32] using nutritional analysis software (COMP EAT 4.0, United Kingdom). The resulting data confirmed that their habitual energy intake remained stable, with macronutrient composition averaging approximately 15 % protein, 60 % carbohydrate, and 25 % fat. Physical activity and dietary habits were monitored throughout the study beyond the initial questionnaire, using in-person interviews during exercise sessions and regular phone calls. This approach ensured adherence to the prescribed regimen and minimized the potential confounding effects of uncontrolled daily physical activity and dietary variations on study outcomes.

#### Statistical analysis

All data are presented as mean ± standard deviation (SD). The Shapiro-Wilk test was employed to verify the normality of pre- and post-intervention values for all dependent variables. A two-way mixed-design ANOVA [3 (groups) × 2 (time points)] was conducted to examine group × time interactions and main effects. When a significant *F*-value was observed, pairwise comparisons were performed using the Bonferroni *post hoc* adjustment. Effect sizes (ES) were calculated using Hedges’ *g*, interpreted as trivial (<0.20), small (0.20–0.59), moderate (0.60–1.19), large (1.20–1.99), or very large (≥2.00), alongside 95 % confidence intervals (CIs) [33]. Statistical significance was set at p<0.05.

## Results

**Fig. 2:**
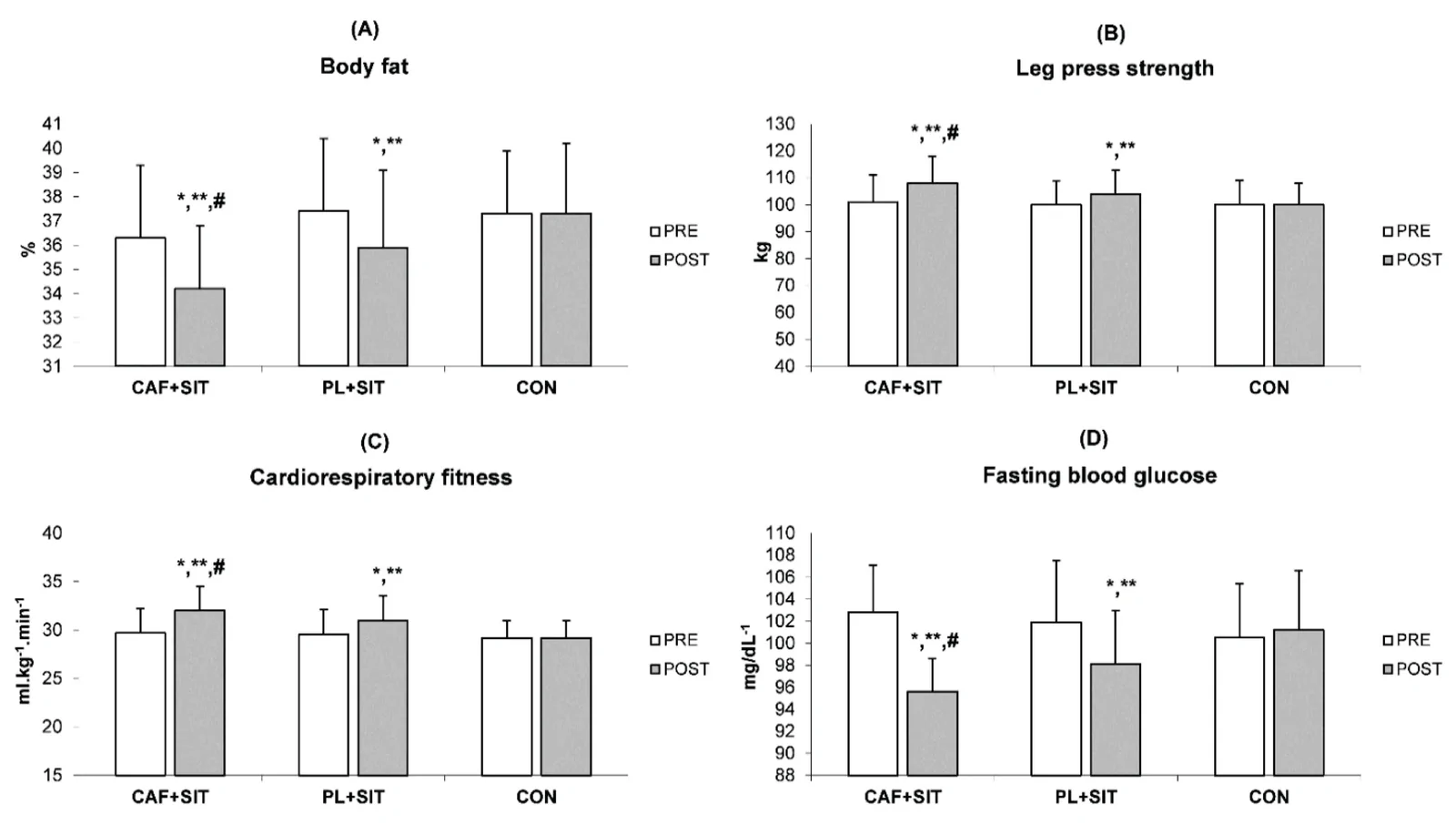
Pre to post-training changes in the (**A**) body fat percent, (**B**) lower body strength, (**C**) cardiorespiratory fitness, and (**D**) fasting blood glucose for the groups (mean ± SD). * Significant differences compared with the PRE (p<0.05), ** Significant differences compared with the CON (p<0.05), ^#^ Significant differences compared with the PL+SIT (p<0.05).

**Fig. 3:**
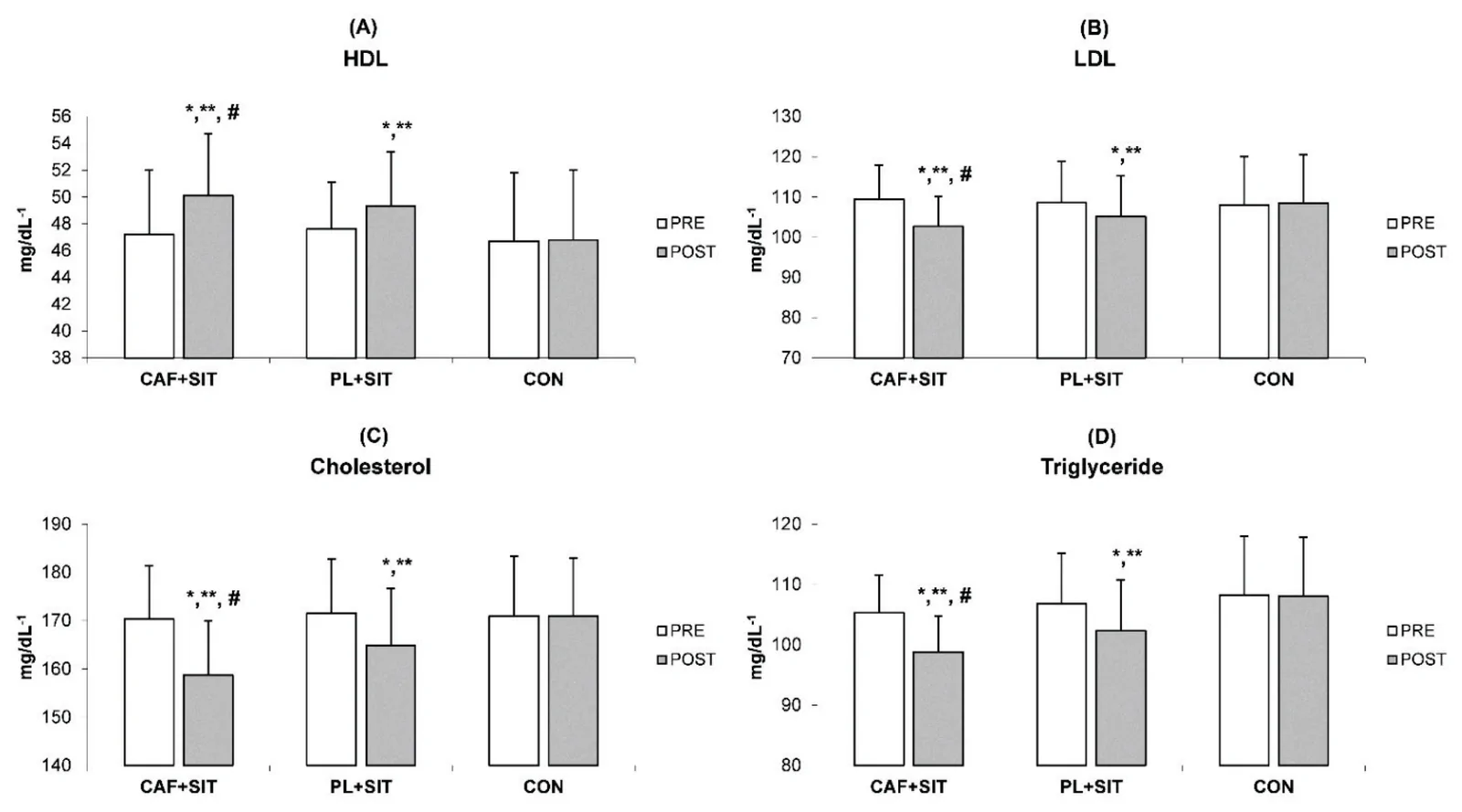
Pre to post-training changes in the (**A**) HDL, (**B**) LDL, (**C**) cholesterol, and (**D**) triglyceride for the groups (mean ± SD). * Significant differences compared with the PRE (p<0.05), ** Significant differences compared with the CON (p<0.05), ^#^ Significant differences compared with the PL+SIT (p<0.05).

**Fig. 4:**
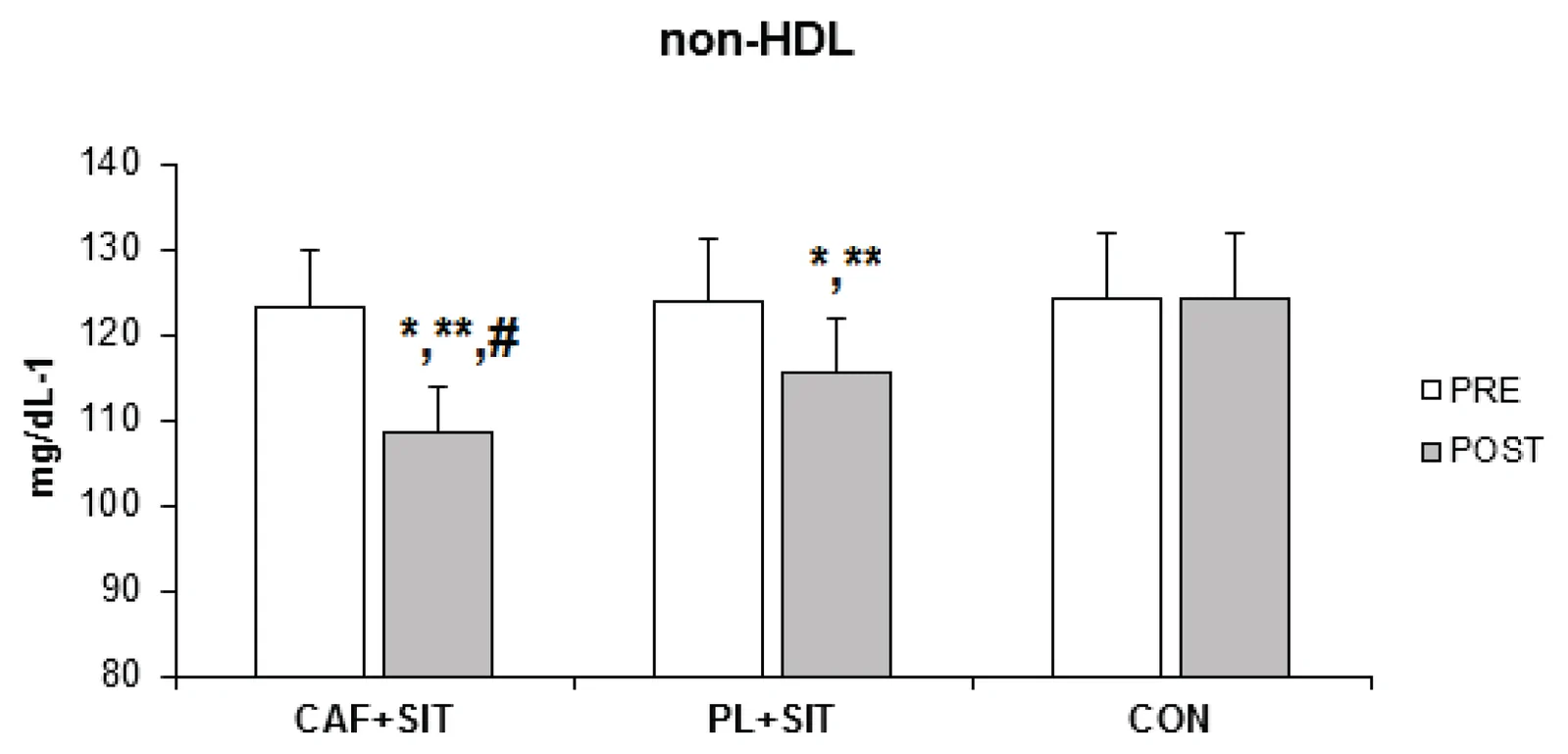
Pre to post-training changes in the non-HDL for the groups (mean ± SD). * Significant differences compared with the PRE (p<0.05), ** Significant differences compared with the CON (p<0.05), ^#^ Significant differences compared with the PL+SIT (p<0.05).

At baseline, no significant differences were observed among the groups in any of the measured variables (p>0.05). All participants assigned to the intervention (i.e., CAF+SIT and PL+SIT) completed every scheduled training session without any attrition or injury occurrence. Additionally, none of the participants reported feelings of anxiety, excessive stimulation, or any adverse responses associated with CAF ingestion. After the 8 weeks’ intervention, no significant changes were observed in the CON group across any of the assessed variables (Figs 2, 3, and 4). In contrast, both the training groups demonstrated significant improvements relative to the CON group in all measured outcomes (p=0.001) (Figs 2, 3, and 4). Within-group analyses further revealed significant pre to post intervention differences in both training groups (p=0.001), with effect sizes ranging from small to large (Table 2). In addition, the CAF+SIT group indicated more adaptations than the PL+SIT group (group × time interaction) in fat mass reduction (standard mean difference (SMD)=−0.58, 95 % CI=−1.44 to 0.31, Small difference, p=0.017), strength gains (SMD=0.36, 95 % CI=−0.54 to 1.22, Small difference, p=0.023), cardiorespiratory fitness improvements (SMD=0.41, 95 % CI=−0.49 to 1.28, Small difference, p=0.014), fasting glucose levels (SMD=−0.57, 95 % CI=−1.44 to 0.35, Small difference, p=0.035), HDL (SMD=0.18, 95 % CI=−0.70 to 1.05, Trivial difference, p=0.039), LDL (SMD=−0.27, 95 % CI=−1.14 to 0.62, Small difference, p=0.044), cholesterol (SMD=−0.53, 95 % CI=−1.39 to 0.33, Small difference, p=0.048), triglyceride (SMD=−0.48, 95 % CI= −1.34 to 0.43, Small difference, p=0.050) and non-HDL (SMD=−1.14, 95 % CI=−2.09 to −0.20, Moderate difference, p=0.033) after the 8-week intervention.

## Discussion

This study investigated whether SIT combined with CAF supplementation elicits greater physiological adaptations than SIT alone with a PL in obese men, focusing on health-related outcomes. Following the 8-week intervention, both the CAF + SIT and PL + SIT groups demonstrated favorable improvements; however, ingestion of CAF at a dosage of 6 mg·kg^−1^ approximately one hour before each training session produced more pronounced adaptive responses in the obese participants. These findings suggest that CAF may potentiate SIT-induced physiological adaptations, offering a feasible, low-cost strategy to enhance body composition and metabolic health within this population.

Our findings align with previous studies demonstrating that various modalities of exercise – particularly SIT – effectively promote reductions in adiposity among overweight and obese individuals [3,6,38]. The primary mechanism underlying fat-mass reduction following SIT appears to involve enhanced lipolytic activity triggered by elevated catecholamine and growth hormone concentrations, which synergistically stimulate hormone-sensitive lipase and promote mitochondrial biogenesis within skeletal muscle [34]. Notably, participants consuming CAF before each SIT session exhibited more pronounced adaptations than those receiving the PL, emphasizing the potentiating role of caffeine’s thermogenic and lipolytic properties in augmenting fat oxidation [35]. Previous investigations consistently identify CAF as a potent ergogenic and metabolic supportive for adiposity reduction [12,15], with evidence indicating that CAF supplementation elevates resting energy expenditure, increases fatty-acid mobilization, and sympathetic drive, increased norepinephrine release, and subsequent upregulation of β-adrenergic-mediated lipolysis [36], thereby collectively facilitating reductions in fat mass.

The present findings demonstrate that 8 weeks of SIT produced improvements in strength performance which is in line with evidence attributing SIT-induced strength enhancements to neuromuscular adaptations facilitating improved force production [19]. SIT has been shown to enhance neuromuscular function by increasing motor unit recruitment efficiency, improving intra- and inter-muscular coordination, and optimizing elastic energy utilization [8,37,38] collectively clarify the observed strength improvements. Moreover, participants supplemented with CAF exhibited greater strength gains than their PL counterparts that clarify the ergogenic effects of CAF-induced augmentation of motor unit activation and increased sarcoplasmic calcium release during high-intensity contractions [18,19]. CAF also acts as a central adenosine-receptor antagonist, elevating sympathetic outflow and reducing perceptions of pain and fatigue [17,39]. Furthermore, its influence on cortical serotonin activity enhances arousal and central drive [17,20], thereby supporting superior motor fiber recruitment throughout SIT sessions. Consequently, the CAF-supplemented group displayed amplified neural activation and subsequent strength adaptation compared with the PL condition [19].

Recent findings demonstrate that both caffeine and placebo interventions significantly enhanced cardiorespiratory fitness which is in line with prior research showing that various forms of SIT are effective to improve aerobic capacity [5,6,7,37,37,40,41]. The gains in among sedentary obese men likely stem from central and peripheral adaptations [31]. Central changes improve oxygen transport *via* greater cardiac output and blood volume, while peripheral adjustments enhance mitochondrial function and capillary density, improving muscular oxygen utilization [8,31]. CAF intake at 6 mg·kg^−1^ before SIT sessions produced more favorable outcomes, indicating it may amplify aerobic adaptations throughout an 8-week program. Mechanistically, its metabolism into theobromine – a vasodilator – increases oxygen and nutrient delivery to brain and muscle tissue, supporting energy turnover and contractile performance [15,16,19,21]. Paraxanthine, which is another active metabolite, elevates circulating glycerol and free fatty acids, promoting fatty acid oxidation as an energy source [21]. CAF also augments calcium release within muscle fibers, activating glycogen phosphorylase b and enhancing oxidative efficiency [16]. Together, these mechanisms reduce perceived exertion and fatigue, contributing to improved and overall cardiorespiratory fitness after the 8-week intervention in obese men.

Both caffeine and placebo groups showed significant reductions in fasting blood glucose after the 8-week intervention, consistent with evidence supporting interval exercise intervention for improving glucose metabolism in overweight and obese individuals [7,10,42,43]. The observed decline in blood glucose is primarily attributed to exercise-induced mechanisms, including increased glucose transport to active muscle fibers, enhanced activation of protein kinase B and upregulated expression and translocation of glucose transporter leading to augments of muscular glucose uptake for ATP resynthesis [10,42]. Additionally, improvements in adipokine such as irisin and adiponectin levels could influence the insulin sensitivity and lipid oxidation [22,42] resulting in lowering blood glucose after SIT intervention in obese men. Notably, CAF supplementation (6 mg·kg^−1^) before SIT produced greater reductions in blood glucose than PL condition, highlighting caffeine’s metabolic potentiation [12,14]. This effect is likely mediated through adenosine receptor blockade, elevated catecholamine release, and cAMP-driven glycogenolysis, as well as CAF-induced activation of AMP-activated protein kinase, which further enhances GLUT4 translocation and glucose uptake [14]. Collectively, these findings indicate that SIT interventions can significantly improve glycemic control through coordinated molecular and endocrine adaptations, while pre-exercise CAF ingestion further magnifies these benefits by promoting complementary metabolic and hormonal responses in obese men [12].

Both the caffeine and placebo groups demonstrated notable improvements in lipid profiles after 8 weeks of SIT, confirming the positive influence of various exercise protocols on lipid profiles in overweight and obese men [5,6,7,44,45]. These favorable changes likely reflect enhanced oxidative metabolism and the upregulation of lipid-regulating genes such as *PPARγ* and *PGC11α* in skeletal muscle and adipose tissue, facilitating mitochondrial biogenesis and fatty-acid oxidation [46,47]. In addition, SIT is thought to increase lipoprotein lipase activity, accelerating triglyceride hydrolysis and improving plasma lipid distribution [7,44]. CAF ingestion (6 mg·kg^−1^) before SIT appeared to further augment these adaptations, producing greater reductions in total cholesterol and triglycerides and a more favorable HDL and LDL as well as non-HDL changes compared with PL [15]. The observed metabolic effects may be partly explained by caffeine’s activation of β3-adrenergic receptors in adipose tissue, a mechanism well-documented in the literature [13–15]. These enhanced effects are presumed to arise from caffeine’s role in stimulating catecholamine-driven lipolysis through β-adrenergic receptor activation, upregulating hormone-sensitive lipase, and initiating cyclic adenosine monophosphate (cAMP) signaling [13]. Such processes may improve fatty-acid mobilization and oxidation, while CAF-induced activation of AMP-activated protein kinase could inhibit acetyl-CoA carboxylase and further stimulate lipid catabolism and mitochondrial efficiency [35]. However, these proposed mechanisms remain speculative, as this study did not directly measure molecular or enzymatic markers of lipid metabolism. Thus, while SIT with CAF produced beneficial alterations in circulating lipid profiles, future research is warranted to elucidate the underlying biochemical pathways and confirm the mechanistic role of CAF in promoting lipid profiles in obese populations.

## Limitation

This study has several limitations that merit thorough discussion. Primarily, the number of included subjects in each group was relatively low, which inherently affects the study’s power; however, we conducted an *a priori* analysis confirming that this sample size appears adequate for our objectives. Secondly, the findings derived from this investigation are specific to male subjects, and additional research is necessary to ascertain whether these results can be reliably transferred to female populations due to known physiological variances. Third, this study is the lack of investigation into caffeine’s effects on participants’ perception of the placebo effect. Caffeine consumption can lead to increased alertness and heart rate, which may act as an interfering factor in evaluating the effectiveness of placebos. In other words, participants might detect a difference between the active drug and the placebo due to the heightened alertness caused by caffeine, potentially influencing the study’s results. Therefore, exploring this aspect in future studies can contribute to a more accurate understanding of caffeine’s impact on treatment response. Another limitation of this study is the absence of waist circumference measurements, which are commonly used to assess central adiposity and metabolic risk. The lack of waist circumference data limits our ability to evaluate abdominal fat and future studies should include this variable measurement for a more comprehensive assessment. Taken together, we view our current findings as preliminary, underscoring the need for subsequent work to either refute or offer further support for these outcomes.

## Conclusions

Our study suggests that participating in SIT is an effective approach for enhancing physiological changes associated with men’s health in obese individuals. Additionally, incorporating CAF supplementation may promote further adaptations, leading to reductions in fat mass, enhancements in strength, improvements in cardiorespiratory fitness, and more effective regulation of fasting glucose levels and lipid profiles. Based on these findings, the use of CAF as an ergogenic aid is recommended to enhance physiological adaptations in obese men with aiming to enhance overall health.

## Figures and Tables

**Table 1:** Participants’ characteristics (mean ± SD).

Variables	CAF+SIT	PL+SIT	CON
*Age (y)*	43.4 ± 1.3	44.1 ± 2.2	43.9 ± 1.8
*Height (cm)*	176.4 ± 3.3	177.1 ± 2.5	175.4 ± 2.9
*Body weight (kg)*	97.4 ± 4.6	98.9 ± 3.7	98.4 ± 4.1
*BMI (kg/m* *^2^* *)*	32.5 ± 1.1	32.3 ± 1.4	33.6 ± 1.8

**Table 2:** ESs with 95 % CI for the variables in the CAF+SIT and PL+SIT groups from pre- to post-training.

Variable	Group	Baseline value	Hedges’ g with 95 % CI	SMD with 95 % CI *CAF+SIT vs. PL+SIT*
***Body fat***	CAF+SIT	36.3 ± 3.1	−0.70 (−1.61 to 0.20) Moderate	−0.58 (−1.44 to 0.34) Small
*(%)*	PL+SIT	37.4 ± 3.0	−0.46 (−1.35 to 0.43) Small	
***Strength***	CAF+SIT	101 ± 10	0.62 (−0.27 to 1.52) Moderate	0.36 (−0.54 to 1.22) Small
*(kg)*	PL+SIT	100 ± 9	0.46 (−0.43 to 1.35) Small	
***Cardiorespiratory***	CAF+SIT	29.7 ± 2.5	0.88 (−0.04 to 1.80) Moderate	0.41 (−0.49 to 1.28) Small
*(ml.kg* *^−1^* *.min* *^−1^* *)*	PL+SIT	29.5 ± 2.6	0.56 (−0.34 to 1.45) Small	
***Fasting blood glucose***	CAF+SIT	102.8 ± 4.3	−1.66 (−2.68 to −0.65) Large	−0.57 (−1.44 to 0.35) Small
*(mg/dl* *^−1^* *)*	PL+SIT	101.9 ± 5.6	−0.70 (−1.60 to 0.21) Moderate	
***HDL***	CAF+SIT	47.2 ± 4.8	0.62 (−0.30 to 1.49) Moderate	0.18 (−0.70 to 1.05) Trivial
*(mg/dl* *^−1^* *)*	PL+SIT	47.6 ± 3.5	0.43 (−0.46 to 1.31) Small	
***LDL***	CAF+SIT	109.4 ± 8.5	−0.80 (−1.71 to 0.12) Moderate	−0.27 (−1.14 to 0.62) Small
*(mg/dl* *^−1^* *)*	PL+SIT	108.6 ± 10.2	−0.33 (−1.21 to 0.55) Small	
***Cholesterol***	CAF+SIT	170.3 ± 11.1	−0.99 (−1.92 to 0.06) Moderate	−0.53 (−1.39 to 0.33) Small
*(mg/dl* *^−1^* *)*	PL+SIT	171.5 ± 11.3	−0.56 (−1.45 to 0.34) Small	
***Triglyceride***	CAF+SIT	105.3 ± 6.3	−1.01 (−1.94 to 0.08) Moderate	−0.48 (−1.34 to 0.43) Small
*(mg/dl* *^−1^* *)*	PL+SIT	106.8 ± 8.4	−0.51 (−1.40 to 0.38) Small	
***Non-HDL***	CAF+SIT	123.1 ± 6.9	−2.27 (−3.40 to 1.15) Very large	−1.14 (−2.09 to −0.20) Moderate
*(mg/dl* *^−1^* *)*	PL+SIT	123.9 ± 7.2	−1.19 (−0.24 to 2.14) Moderate	
